# Ferulic acid‐impregnated sodium alginate–pectin biopolymer film for active packaging and shelf‐life extension of potato chips

**DOI:** 10.1002/jsfa.70672

**Published:** 2026-04-21

**Authors:** Shaik Sadiya, Venkata Giridhar Poosarla, Navyasri Nandavarapu, Aparna Ramadoss, Nagaveni Shivshetty

**Affiliations:** ^1^ Department of Life Sciences GITAM School of Science, GITAM (Deemed to be University) Visakhapatnam India

**Keywords:** active films, ferulic acid, antioxidant properties, potato chips, shelf‐life extension

## Abstract

**BACKGROUND:**

This study explores the development of active films incorporating ferulic acid (FA), a natural antioxidant, at concentrations of 0 (F1), 2.5 (F2), and 5 g L^−1^ (F3) into sodium alginate (SA; 13 g kg^−1^) and pectin (P; 10 g kg^−1^) matrix. The developed SA–P–FA films (F1, F2, and F3) were investigated for their physical, optical, mechanical, barrier, antioxidant, structural, and morphological properties. Incorporation of FA into the films may improve the antioxidant, barrier, and resistance to oxidative deterioration properties of lipid‐rich foods.

**RESULTS:**

Among the developed films, the highest antioxidant activity (AA) of 90%, tensile strength (TS) of 2.9 MPa, and low water vapor permeability (WVP) of 5.4 × 10^−11^ g m^−1^ s^−1^ Pa^−1^ were observed for the F3 film, whereas F1 exhibited lower AA (30%) and TS (1.8 MPa), and higher WVP (8.73 × 10^−11^ g m^−1^ s^−1^ Pa^−1^), indicating the potential of FA. The F3 film also exhibited a high water contact angle of 116.7°, indicating FA hydrophobicity and crosslinking ability, enhancing film compactness. Furthermore, the shelf life of potato chips was studied with three sets of samples (unpacked chips (control), F1, and F3 film‐wrapped chips). The chips packed in F3 film exhibited low water activity (0.54), peroxide value (6.5 mEq kg^−1^), AA (34%), and crispness by maintaining sensory attributes until day 10.

**CONCLUSION:**

These findings demonstrate that SA–P–FA films have strong potential as excellent antioxidants and sustainable packaging materials for extending the shelf life of food products prone to oxidative deterioration, such as potato chips. © 2026 The Author(s). *Journal of the Science of Food and Agriculture* published by John Wiley & Sons Ltd on behalf of Society of Chemical Industry.

## INTRODUCTION

Food packaging is a crucial aspect of modern food systems, plastics being widely used as they are versatile, durable, and inexpensive.^1^, ^2^ However, concerns have been raised about the potential health risks associated with certain plastic additives.^3^ Annually, 40 million tons of global plastic waste are generated, leading to environmental effects, including contamination of soil and water bodies and emission of harmful gases during disposal.^4^, ^5^ In addition to these concerns, lipid oxidation is another major issue in foods that contain high lipid content. Foods such as potato chips may develop off‐flavors, rancidity, and deterioration during storage. These problems can be addressed by incorporating natural antioxidants into sustainable, biodegradable active packaging materials that delay oxidative deterioration while maintaining food quality.^6^, ^7^, ^8^

The demand for developing active and biodegradable packaging has increased, including natural biopolymer ingredients such as cellulose, pectin (P), lignin, sodium alginate (SA), starch, chitosan, and gelatin.^9^ SA is an example of a biodegradable polymer derived from the cell walls of brown algae, in the form of magnesium salts of alginic acid, calcium, and sodium.^10^ SA is a linear copolymer composed of (1 → 4)‐*β*‐d‐mannuronic acid blocks (M) and (1 → 4)‐*α*‐l‐guluronic acid (G).^11^ The US Food and Drug Administration (FDA) has declared SA as generally recognized as safe (GRAS).^12^ SA is commonly used in the beverage and food industries as a gelling, thickening, and stabilizing agent in various beverages, sauces, and desserts.^11^

P is another GRAS‐certified polysaccharide, a natural gelling agent derived from fruit and vegetable peels. Its excellent film‐forming ability and flexibility make it a commonly used component in the packaging industry.^13^, ^14^ A combination of natural polymers can enhance the ability to form film, and the combination of SA and P can create a strong and stable base polymer matrix. In the presence of divalent cations, they further develop a three‐dimensional ‘egg‐box’ network with enhanced flexibility and barrier properties.^15^, ^16^

The developed polymer matrix can be further modified by adding active components such as extracts obtained from plants, essential oils, and various phenolic compounds, including ferulic acid (FA), gallic acid, caffeic acid, and tannic acid.^17^ A study incorporating these phenolic compounds into polysaccharide‐based chitosan films containing gallic acid, caffeic acid, and FA reported improved mechanical and barrier properties.^18^ The integration of phenolic compounds, including protocatechuic acid, naringin, and tannic acid, into maize starch‐based films showed enhanced antioxidant properties.^19^ These active ingredients can further enhance a polymer's antioxidant and antimicrobial properties, thereby increasing the shelf life of food products by reducing oxidation and microbial contamination.^17^ FA is a plant‐derived phenolic molecule that has strong antioxidant properties and is GRAS.^20^ It is approved for use in food applications by both the FDA and the European Union (EU) regulations.^21^ It can interact with a polymer matrix through crosslinking to produce stable free radicals.^22^ FA is abundantly present in plants commonly used as antioxidants in the beverage and food industries, contributing to their nutritional profile.^23^ Studies have incorporated FA as an antioxidant in polymer matrices, improving the shelf life of foods such as apples,^24^ pork,^25^ and beef.^26^

Potato chips are widely consumed snacks, but they have a very short shelf life. A major cause of deterioration is lipid oxidation, which is accelerated by oil absorption during frying and results in product spoilage during storage. Studies have explored monitoring lipid oxidation during processing and preserving chips and other fried snacks, particularly by adding natural antioxidants.^27^

The novelty of the study reported here lies in the fact that there are no reports, to the best of our knowledge, on the combination of SA, P, and FA. Various studies have explored the integration of FA in different polymer matrices and their application to food materials. Studies include polyethylene oxide, zein, and FA films applied onto apples, which improved shelf life by preventing water loss and quality deterioration.^24^ Similarly, films made with carboxymethylcellulose, polyethylene glycol, natamycin, and FA applied to Ras cheese reduced its moisture content by maintaining titratable acidity.^28^ Furthermore, films composed of poly[(3‐hydroxybutyrate)‐*co*‐(3‐hydroxyvalerate)], rice straw extracts, and FA reduced the oxidation of pork by extending its shelf life (Table S1).^29^ However, the use of a specific combination of FA with SA and P for high‐lipid‐snack preservation is novel. It addresses a need, particularly in preventing rancidity in potato chips. Since these natural substances have not yet reached their full potential for improving food preservation, there is scope for further research. The study reported here aimed (a) to formulate an active film rich in antioxidants by incorporating FA into SA and P, (b) to characterize the potential of the developed films, and (c) to study the extension of the storage period of potato chips. This aids in the packaging of foods that are highly susceptible to rancidity, particularly those with high lipid content, and extends their shelf life. This can serve as a replacement for conventional packaging, making the film more sustainable.

## MATERIALS AND METHODS

### Chemicals and reagents

Food‐grade chemicals including SA extrapure (91–106%) (sodium polymannuronate), cat. no. 11510SG500, and molecular weight: 216.1212 g mol^−1^ (Finar, Gujarat, India); pure P (minimum of 50% galacturonic acid and high methoxyl content 7%), cat. no. 45057, and molecular weight: 25 000 to 50 000 g mol^−1^ (Sisco Research Laboratory, Maharashtra, India); FA pure (98%), cat. no. 60096, and molecular weight: 194.19 g mol^−1^ (Sisco Research Laboratory, Maharashtra, India); glycerol (Qualigens, Mumbai, India); methanol; and 2,2‐diphenyl‐1‐picrylhydrazyl (DPPH; Sisco Research Laboratory, Maharashtra, India) were purchased. Potato chips were purchased from a local bakery in Visakhapatnam, India.

### Active film preparation

**Figure 1:**
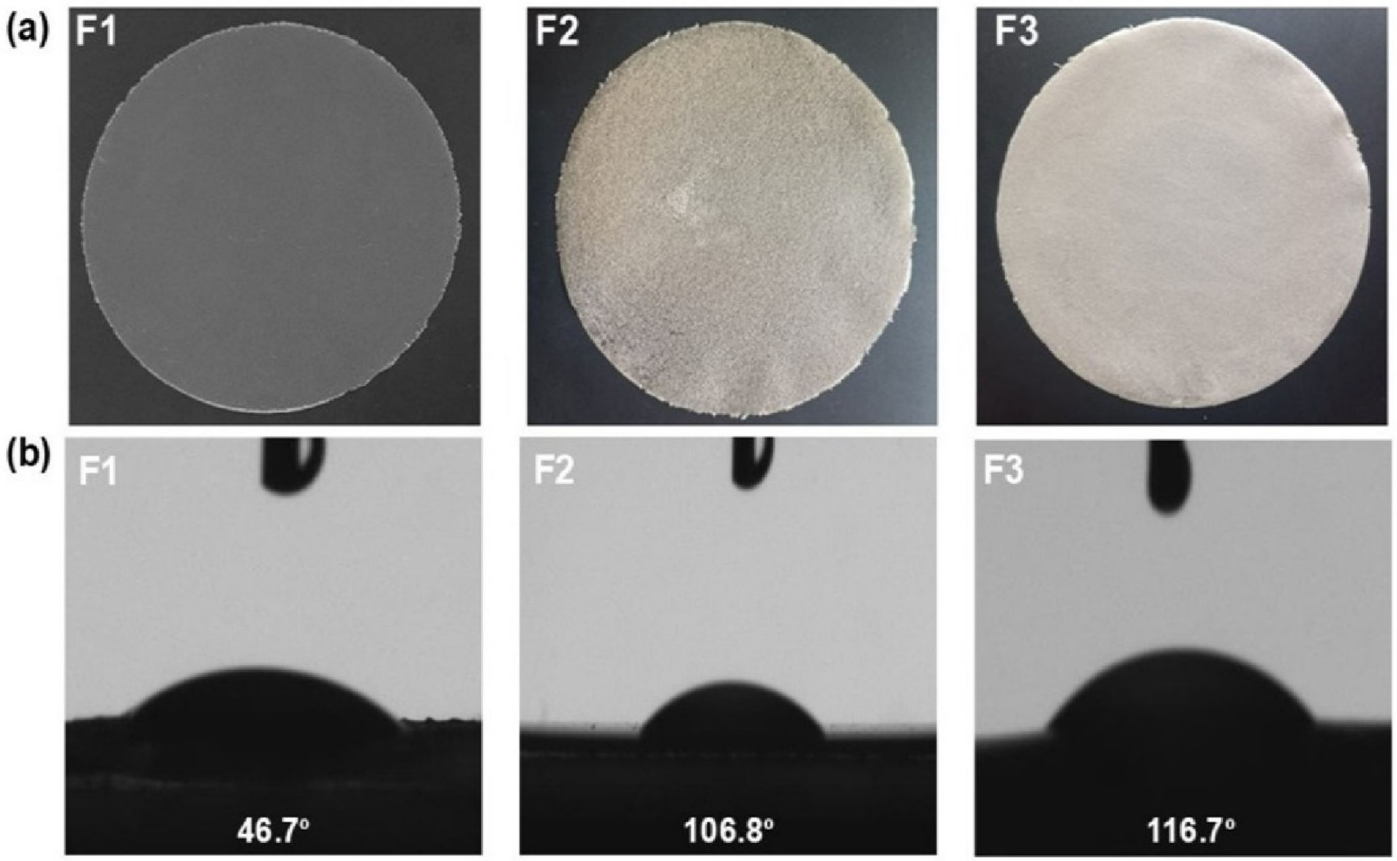
(a) Development of films using SA, P, and FA. The films were poured onto a glass Petri dish (145 mm in diameter). (b) WCA of the developed films. F1, SA + P; F2, SA + P + 2.5 g L^−1^ FA; F3, SA + P + 5 g L^−1^ FA.

The polymer films were prepared by solvent casting, including minor modifications.^30^, ^31^ SA (13 g kg^−1^) and P (10 g kg^−1^) were added simultaneously to a glass beaker containing 100 mL of distilled water (DW), and the mixture was dissolved using a hot plate at 500 rpm and 70 °C for 30 min until the solution became thick and clear. During heating, approximately 10 mL of the film‐formulating solution evaporated, resulting in a thicker solution. Furthermore, different concentrations of FA (0, 2.5, and 5 g L^−1^) were added to the film‐forming solution, followed by glycerol (15 g L^−1^), with constant stirring (500 rpm) to ensure proper blending. Finally, the resultant solution (approximately 90 mL) was poured onto a Petri plate (145 mm in diameter) and dried in a biological oxygen demand chamber for 24 h at 30 °C and 50% relative humidity (RH) until no further change in film weight was observed.^32^ The films were removed from the casted plate after drying and labelled as F1, SA + P; F2, SA + P + 2.5 g L^−1^ FA; F3, SA + P + 5 g L^−1^ FA, as shown in Fig. 1(a).

### Physical characterization of films

#### Thickness

The thickness of the films was measured using a Vernier caliper with a least count of 0.00 mm at three random points, and the average value was determined.^33^

#### Moisture content (MC)

Developed films were conditioned at 30 °C and 50% RH for 48 h before analysis.^34^ The films were cut into (2 × 2 cm^2^) square pieces, and the initial weight was noted. Later, the sample was heated in a hot‐air oven for 3 h at 105 °C, and the final weight was determined. Moisture content was measured in triplicate and expressed as a percentage using Eqn (1)^31^:

(1) $$\mathrm{MC}\;\left(\%\right)=\;\frac{W_{1}-W_{2}}{W_{1}}\times 100$$

where *W*_1_ represents the initial weight of the film and *W*_2_ represents the final weight of the film after drying.

#### Swelling index (SI)

The SI of the films was studied by soaking a film sample (2 × 2 cm^2^) in water for 2 min. The film was weighed to measure and calculate the SI using Eqn (2)^31^:

(2) $$\mathrm{SI}\;\left(\%\right)=\frac{W_{s}-W_{d}}{W_{d}}\times 100$$

where *W*_s_ represents the weight of the swollen film and *W*_d_ the weight of the dry film.

#### Water solubility (WS)

The WS of the films was determined by submerging the film (2 × 2 cm^2^) into DW (10 mL) and keeping it in a shaking incubator (24 h). The film, which remained undissolved, was separated by centrifugation (236 × *g* for 20 min). The film was dried in an oven (105 °C for 24 h) and WS was calculated using Eqn (3)^31^:

(3) $$\mathrm{WS}\;\left(\%\right)=\frac{W_{i}-W_{f}}{W_{i}}\times 100$$

where *W*_i_ indicates the initial weight of the film and *W*_f_ indicates the weight of the dried film.

#### Density

The film density (4 × 1 cm^2^) was directly calculated from the film mass, area, and thickness using Eqn (4). The sample density was measured in triplicate.^31^

(4) $$\text{Density}\;\left(g\;{\mathrm{cm}}^{-3}\right)=\;\frac{M}{A\times d}$$

where *M* represents the mass (g), *A* is the area (cm^2^), and *d* indicates the thickness of the film.

#### Water activity

The water activity (*a*_w_) of the samples was measured using a water activity meter (Novasina, LabSwift‐AW, Japan). The meter was calibrated using suitable standards at 30 °C before analysis. The analysis involved measurement in triplicate.^33^

#### Water vapor permeability (WVP)

The WVP was determined according to American Society for Testing and Materials (ASTM) standard test method E96/96M to determine the amount of water diffused through the developed film.^35^ A glass test tube containing anhydrous calcium chloride was sealed with films and kept in a desiccator containing a saturated sodium chloride solution (30 °C, 75% RH). The weight change of the tubes was recorded every 24 h until the tubes reached a constant weight (0.001 g). WVP was calculated using Eqn (5)^33^:

(5) $$\mathrm{WVP}\;\left(g\;m^{-1}\;s^{-1}\;{\mathrm{Pa}}^{-1}\right)=\frac{\left(m_{1}-m_{0}\right)\times L}{A\times t\times ∆P}$$

where the final and initial weights of the tube are *m*_0_ and *m*_1_, the thickness of the film is *L*, *t* indicates time, and Δ*P* represents the water vapor pressure on both sides of the film.

### Water contact angle (WCA)

To determine the WCA of SA–P–FA films, a contact angle meter (Acam‐HSC 19, Apex Instruments, Kolkata, India) was used, followed by the sessile drop method with minor changes.^36^ A water droplet (7 μL) was dispensed onto the film surface using a glass syringe under controlled conditions (25 °C, 50% RH). The final WCA value represents the average of five measurements.

### Optical properties

To study the transparency and opacity, the films were cut into 4 × 1 cm^2^ and measured using a UV spectrophotometer (Shimadzu UV‐1800, Nakagyo‐Ku, Kyoto, Japan). The transparency of the films was measured at 800 nm and calculated using Eqn (6). To determine the opacity, the films were measured at 670 nm and Eqn (7) was used to calculate the values.^33^

(6) $$\text{Transparency}=\frac{\log\left(\%T_{800}\right)}{x}$$

(7) $$\text{Opacity}=\frac{{\text{Abs}}_{670}}{x}$$

where %*T*₈₀₀ and Abs₆₇₀ correspond to the transmittance at 800 nm and absorbance at 670 nm, respectively, and *x* represents the film thickness (mm).

### Mechanical properties

A micro‐tensile tester (ZT‐MX2‐500N, Imada) was used to evaluate the film tensile strength (TS), elongation at break (EAB), and Young's modulus (YM). All these tests were measured in triplicate.^31^

### Antioxidant activity of films

The free radical scavenging ability of the developed active packaging was tested using the DPPH assay.^37^ To prepare the DPPH solution, 4 mg of DPPH was weighed and dissolved in 100 mL of methanol. The developed films were weighed (6 mg, dry weight) and added to 3 mL of DPPH solution (0.1 mmol L^−1^) in test tubes. The concentration of DPPH solution was selected based on its optimum absorbance range (0.8–1.0 at 517 nm). The tubes were then kept at room temperature in a dark place for 30 min. Based on DPPH discoloration, radical scavenging activity was measured at 516 nm using a UV–visible spectrophotometer (Shimadzu, Nakagyo‐Ku, Kyoto, Japan). The antioxidant activity (AA) was expressed as a percentage using Eqn (8), where the control value was taken from a plain DPPH solution:

(8) $$\mathrm{AA}\;\left(\%\right)=\frac{A_{0}-A_{T}}{A_{0}}\times 100$$

where *A*_0_ represents the absorbance of the control while *A*_T_ represents the absorbance of the film sample.

### Release of FA from films

To determine the amount of FA released from the developed films, release studies based on total polyphenol content (TPC) were accordingly performed for SA–P–FA films.^38^ In this study, 96% ethanol was used as a food simulant in accordance with EU law.^39^ The developed films (F1, F2, and F3) were weighed (100 mg) and added to an amber bottle containing ethanol (40 mL) at 25 °C for 24 h in a shaking incubator. To determine the amount of FA released into the food simulant, 0.25 mL of the sample was mixed with 1.25 mL of 10% (v/v) Folin–Ciocalteu reagent. The combined solution was vortexed and incubated in the dark at 25 °C for 5 min. After incubation, 1 mL of sodium carbonate solution (75 g L^−1^) was added to the reaction mixture, which was then incubated in the dark for 60 min. The absorbance was measured at 765 nm using a UV–visible spectrophotometer. TPC was quantified using the FA standard curve, and the values were calculated using Eqn (9):

(9) $$\mathrm{TPC}\;\left(\mathrm{mg}\;g^{-1}\right)=\frac{C\times V}{m}$$

where *C* is the FA concentration determined from the calibration curve (mg mL^−1^), *V* is the volume of the dissolved film solution (mL), and *m* is the weight of the film sample (g).

### Structural and morphological characteristics of films

#### Fourier transform infrared (FTIR) spectroscopy

Functional groups in the SA–P–FA films were investigated with attenuated total reflectance FTIR spectroscopy (Bruker Alpha‐II, Germany). The spectra were measured over a wavenumber range of 4000 to 500 cm^−1^, with a resolution of 4 cm^−1^, with 32 scans recorded for each sample.^40^

#### X‐ray diffraction (XRD)

The crystallinity of the SA–P–FA film was determined using a powder XRD device (Bruker D8 Advance, USA) with a Cu K*α* radiation source (1.5418 Å) at 40 mA. The scan time was 0.5 s per step, over a 2*θ* range of 0.02° with a voltage of 40 kV.^33^ To evaluate the different peaks obtained from the XRD patterns, TOPAS software (version 7.0) was used (Total Pattern Solution, Bruker™ Company, Karlsruhe, Germany). To calculate the degree of crystallinity, the area of the peak was divided by the total area of the diffraction curve.^41^

#### Field‐emission scanning electron microscopy (FE‐SEM)

The F1 film without FA incorporation and the F3 film with FA were examined using FE‐SEM (Tescan, Mira, Brno, Czech Republic) to assess surface morphology. A small portion of sample was cut for imaging, followed by the application of a 10 nm thin layer using a LUXOR metal coater under an argon atmosphere. A double‐sided carbon tape was used to mount the film, and an accelerating voltage of 3 keV was applied to capture its surface structure.^40^

### Shelf‐life studies

#### Packaging of potato chips

Three film formulations (F1, F2, and F3) were initially developed and characterized to evaluate the influence of FA concentration on the physicochemical and functional properties of the films. To assess the performance of the developed active films, shelf‐life studies were conducted using premium‐grade potato chips, which are globally popular as a fried snack, have a high lipid content, and are susceptible to rancidity. The chips used for analysis were purchased from a local bakery in Visakhapatnam, India, and fried in sunflower oil (Saffola, India) at 180 °C. The initial quality of the potato chips on day 0 was considered the baseline and was defined based on physicochemical and sensory attributes. Since F3 (5 g L^−1^ FA) exhibited superior properties, including enhanced mechanical strength, reduced WVP, and higher AA, F3 (*n* = 18) was selected as the most promising formulation for the shelf‐life study. Additionally, to assess the impact of FA, chips were packed in FA‐free films (F1) (*n* = 18), while control samples remained unpacked (*n* = 18). All the samples were stored at 30 °C, with 50% RH, and the samples were kept in dark conditions inside a biological oxygen demand chamber. Since F2 showed reduced mechanical strength and antioxidant properties compared to F3, it was excluded from the application study to maintain a clear comparison between F3 (with FA), F1 (without FA), and unpacked. The packets (F1 and F3) were heat‐sealed and opened on each sampling day before analysis. A comprehensive analysis was performed on all groups, with a primary focus on rancidity evaluation. This study develops antioxidant‐rich active films loaded with FA to minimize oxidation of potato chips, thereby creating stable active film applications for food preservation.^42^

#### Moisture content

The MC of potato chips was analyzed in a hot‐air oven, where 5 × 10^−3^ kg of potato chips was weighed and dehydrated at 105 °C for 4 h until the weight became constant. The MC of the chips was calculated using Eqn (10), and the results were expressed as percentages. All measurements were performed in triplicate.^43^

(10) $$\mathrm{MC}\;\left(\%\right)=\;\frac{W_{1}-W_{2}}{W_{1}}\times 100$$

where the initial weight of the sample is *W*_1_ and *W*_2_ is the final weight of the sample.

#### Water activity

A water activity meter (Novasina, LabSwift‐AW, Japan) was used to measure *a*_w_ of potato chips. The samples were kept in a water activity meter until the readings were constant. The readings for all the samples were taken in triplicate.^44^

#### Peroxide value (PV)

The PV was determined by measuring the amount of peroxide oxygen formed per kilogram of oil or fat, expressed in millimoles or milliequivalents per kilogram. To determine the PV, 1 × 10^−3^ kg of the sample was mixed with a 3:2 ratio of glacial acetic acid and chloroform, and boiled for 30 s. Then, 20 mL of 50 g L^−1^ potassium iodide, which contains 0.3 × 10^−3^ kg iodine and 50 mL DW, was added. The titration of the mixture was performed using sodium thiosulfate (Na_2_S_2_O_3_, 0.01 mol L^−1^) until the yellow color had completely faded. Then 5 × 10^−4^ L of starch (100 g L^−1^) was added to the mixture as an indicator, and the mixture was further titrated until the blue color had disappeared.^45^ The PV was calculated based on the amount of thiosulfate used with Eqn (11):

(11) $$\mathrm{PV}\;\left(\text{milliequivalent peroxide}/\mathrm{kg}\;\text{sample}\right)=\;\frac{S\times N\times 1000}{g\;\text{sample}}\;\mathrm{mEq}\;{\mathrm{kg}}^{-1}$$

where *S* represents the volume of Na_2_S_2_O_3_ (test‐blank) and *N* is the normality of Na_2_S_2_O_3_.

#### Antioxidant content

The antioxidant compounds were extracted from potato chips by combining 3.5 × 10^−6^ m^3^ of ethanol with crushed chips (3 g), and their antioxidant content was measured accordingly, with minor modifications.^46^ The extract (3.5 × 10^−6^ m^3^) was combined with 3 × 10^−6^ m^3^ of DPPH solution and incubated in the dark for 30 min. The absorbance was then recorded at 516 nm using a UV–visible spectrophotometer. Equation (12) was used to calculate the antioxidant content of potato chips, with the control value obtained from a plain DPPH solution:

(12) $$\mathrm{AA}\;\left(\%\right)=\frac{A_{0}-A_{T}}{A_{0}}\times 100$$

where *A*_0_ is the absorbance of the control sample and *A*_T_ is the absorbance of the film sample.

#### Hardness

The crispiness of the potato chips was analyzed using a texture analyzer (TA‐CT3, Brookfield Engineering Laboratories, Inc., MA, USA) to measure hardness. The chips were placed under a 2 mm radius cylindrical probe (TA‐AACC36), with a constant compression speed of 2 mm s^−1^, a trigger force of 2.94 N, and a deformation distance of 0.5 mm. The analysis was performed using the dual compression method, with a 15 s interval between the two compression cycles.^47^

#### Sensory evaluation

Sensory evaluation was conducted on every 0, 2, 4, 6, 8, and 10 days of storage for three groups of potato chip samples (unpacked, F1, F3). The quality of potato chips was assessed by a panel consisting of 10 individuals. The panelists were students from the Department of Life Sciences at GITAM School of Science, GITAM (Deemed to be University). Before the analysis, all the panelists were semi‐trained and briefed on the sensory attributes and evaluation criteria to ensure uniformity throughout the evaluation.^48^ The sensory attributes, including color, aroma, texture, taste, and appearance, were assessed using a 9‐point hedonic scale, where 1 indicates ‘dislike extremely’, 5 indicates ‘neither like nor dislike’, and 9 indicates ‘like extremely’.^49^ Although the panelists were selected from the same department, care was taken to minimize potential bias by providing standardized instructions and conducting random sample coding under controlled conditions, including warm white light and ambient temperature (30 ± 2 °C).

### Statistics

Statistical analyses were conducted using OriginPro 8.5. Tukey's honestly significant difference (HSD) test was used with one‐way analysis of variance (ANOVA) to evaluate differences among the films (F1, F2, and F3) for the measured film characterization parameters. The Bonferroni test was used to perform a two‐way ANOVA to examine the effects of film type and storage time of potato chips. The difference was considered statistically significant at *P* < 0.05.

## RESULTS AND DISCUSSION

This study develops active, biodegradable films with improved functional properties that further help preserve high‐lipid‐content foods, such as potato chips. The results demonstrate that FA functions both as an active antioxidant and as a molecular modifier within the SA–P film matrix. FA incorporation induced structural changes in films, improving their properties and extending the shelf life of wrapped potato chips.

### Film characterization

#### Thickness

As the FA concentration increases in films F1, F2, and F3 (0, 2.5, and 5 g L^−1^), the film thickness significantly increases (*P* < 0.05) from 0.37 ± 0.03 to 0.47 ± 0.08 mm (Table 1). The increase in thickness values upon adding FA indicates the development of strong molecular interactions, primarily through hydrogen bonding between phenolic hydroxyl groups and polymer chains, resulting in matrix densification, reduced hydrophilicity, and improved mechanical and barrier properties.^50^ Similar findings were reported in a previous study,^51^ which demonstrated that thickness varies with the concentration in the film formulation. Furthermore, the film's increased thickness helps water vapor to diffuse slowly through the film matrix, further contributing to the decrease in WVP values.^52^

**Table 1:** Characterization of developed active films

Film	Physical properties						Barrier properties	Optical properties		Bioactive properties	
	*T* (mm)	MC (%)	SI (%)	WS (%)	*D* (g cm^−3^)	WA	WVP (10^−11^ g m^−1^ s^−1^ Pa^−1^)	Ty (%)	*O* (A/mm)	AA (%)	TPC (mg g^−1^)
F1	0.37 ± 0.03^a^	35 ± 0.3^a^	214 ± 0.4^a^	93 ± 0.9^a^	0.061 ± 0.01^a^	0.59 ± 0.03^a^	8.73 ± 0.2^a^	0.72 ± 0.02^a^	0.37 ± 0.3^a^	30 ± 2.5^a^	0.42 ± 0.09^a^
F2	0.45 ± 0.04^b^	30 ± 0.5^a^	205 ± 0.7^b^	83 ± 0.5^b^	0.067 ± 0.01^b^	0.57 ± 0.03^b^	6.35 ± 0.4^b^	0.41 ± 0.03^b^	0.85 ± 0.14^b^	85 ± 1.1^b^	0.77 ± 0.05^b^
F3	0.47 ± 0.08^b^	25 ± 0.1^b^	167 ± 0.7^c^	74 ± 0.6^c^	0.072 ± 0.01^b^	0.55 ± 0.04^b^	5.4 ± 0.3^c^	0.28 ± 0.03^c^	1.16 ± 0.3^c^	90 ± 0.95^b^	1.4 ± 0.05^c^

F1, SA + P; F2, SA + P + 2.5 g L^−1^ FA; F3, SA + P + 5 g L^−1^ FA. Data are presented as mean ± SD, with all measurements made in triplicate. Significant differences (*P* < 0.05) are represented by different letters according to Tukey's HSD test.

AA, antioxidant activity; *D*, density; FA, ferulic acid; MC, moisture content; *O*, opacity; P, pectin; SA, sodium alginate; SI, swelling index; *T*, thickness; TPC, total polyphenol content; Ty, transparency; WA, water activity; WS, water solubility; WVP, water vapor permeability.

#### Moisture content

The MC indicates the moisture retained in the developed films (Table 1). The F1 film containing SA and P without FA shows more sensitivity towards moisture when compared with films containing 2.5 and 5 g L^−1^ FA. The MC was reduced in F2 and F3 films with the addition of FA from 30 ± 0.5% to 25 ± 0.1% compared to the control (F1), which was 35 ± 0.3% (*P* < 0.05). The reduction of MC in the film was attributed to the crosslinking of the polymer matrix and FA.^53^ This was further supported by the FTIR analysis, which clearly indicates the presence of characteristic peaks of FA contributing to the hydrophobicity of the film. These findings were in agreement with a previous study on SA edible films, which were incorporated with FA. The films exhibited a reduction in moisture from 89% to 87%.^51^

#### Swelling index

The SI of the films decreases as the FA concentration increases (Table 1). The F1 film showed an SI of 214 ± 0.4%, compared with films containing FA (F2 and F3), which exhibited lower indices of 205 ± 0.7% and 167 ± 0.7%, respectively (*P* < 0.05). This phenomenon was attributed to the interaction between FA and the polymer matrix, leading to fewer hydrophilic sites and reduced water uptake.^50^, ^54^ A previous study, based on chitosan–alginate films, crosslinked with FA, showed that the SI decreased from 272% to 175%.^50^

#### Water solubility

The WS was higher for F1 film (93 ± 0.9%) than for F2 and F3 (83 ± 0.5 and 74 ± 0.6%) due to the hydrophobic property of FA (*P* < 0.05) (Table 1). The solubility of the film decreases (partial solubility) with increasing concentration due to the development of a stable network in the film matrix with FA, which prevents interaction with hydrogen molecules.^53^ The partial solubility helps the film to be stable even at high‐humidity conditions by maintaining the quality of the food product without compromising the biodegradability of the film.^52^, ^55^ In a previous study, films made with chitosan and SA incorporated with FA showed a similar decreasing trend.^50^

#### Density

The density of the films increased with the increase in concentration of FA (Table 1), where the control film (F1) exhibited a density of 0.061 ± 0.01 g cm^−3^. The density increases to 0.067 ± 0.01 g cm^−3^ in F2 and 0.072 ± 0.01 g cm^−3^ in F3 due to the incorporation of FA (*P* < 0.05). The low density of F1 was attributed to its molecular structure, which included free volume in the SA and P matrix. As the F2 and F3 films contain FA, the density gradually increases due to the formation of strong interactions between the polymer matrix and FA. These interactions increase density by reducing the film's free volume.^56^ These results are in agreement with those of a previous study, which found that SA films incorporating a mixed probiotic culture exhibited an increase in density (1.17 to 1.91 g cm^−3^).^57^

#### Water activity

A decline in *a*_w_ was observed from F1 to F3 (0.59 ± 0.03 to 0.55 ± 0.04), due to the increase in the concentration of FA (*P* < 0.05) (Table 1). The *a*_w_ value for F3 was reported below 0.6 threshold, which inhibits microbial growth.^58^ This decline was observed due to the hydrophobic nature of FA, which has benefits such as enhanced microbial stability and prolonged shelf life for food products.^59^ In a previous study, films made from potato byproducts and gallic acid exhibited a decrease in *a*_w_ from 0.0893 to 0.0851, which supports the current research.^60^

#### Water vapor permeability

As the FA concentration increases, the WVP of the films decreases, as presented in Table 1. The F1 film presented a WVP of 8.73 ± 0.2 × 10^−11^ g m^−1^ s^−1^ Pa^−1^, while the FA‐incorporated films (F2 and F3) showed lower values of 6.35 ± 0.4 and 5.4 ± 0.3 × 10^−11^ g m^−1^ s^−1^ Pa^−1^ (*P* < 0.05). The WVP values of various commercial packaging materials are presented in Table S2. A suitable WVP range for food packaging is 0.1 to 13.57 g mm m^−2^ h^−1^ kPa^−1^.^61^ The WVP of F3 film lies in the above range (0.19 g mm m^−2^ h^−1^ kPa^−1^) after unit conversion, which further supports its suitability for lipid‐rich food application. These improved barrier properties can be due to the presence of FA in the SA–P matrix. It enhances crosslinking between the film matrix and FA, thereby reducing the number of hydrogen‐bonding sites and making the polymer network more compact and stable.^62^ A similar trend toward reduced film thickness has been reported in previous studies, in which impregnation with FA improved film barrier properties.^53^, ^62^

#### Water contact angle

The WCA indicated the surface wettability of the developed active packaging. The F1, F2, and F3 films showed an increasing trend in WCA (Fig. 1(b)). The F1 film exhibited a low WCA of 46.7 ± 1.7°, whereas incorporation of FA increased the WCA. As the FA concentration increases from F2 to F3, the WCA increases from 106.8 ± 2° to 116.7 ± 1.5°, demonstrating enhanced hydrophobicity (*P* < 0.05). The hydrophobic behavior of F2 and F3 films was attributed to the presence of FA, which is insoluble in water due to its surface roughness and the hydrophobic functional groups (aromatic bonds of C=C and carboxylic acid of C—O stretching).^63^, ^64^ These results are in agreement with a previous study where chitosan films were prepared by incorporating FA and flaxseed oil nanoemulsions, which showed an increase in WCA from 82.80° to 91.23°.^65^ This difference may be attributed to variations in the polymer matrix and the distribution of FA within the film structure.

#### Transparency and opacity

The transparency of the films decreases as their opacity increases, as shown in Fig. 1. The control film (F1) exhibited high transparency (0.72 ± 0.02%), whereas the F3 film with FA showed lower transparency (0.28 ± 0.03%) (*P* < 0.05) (Table 1). Correspondingly, the opacity of F1 was low (0.37 ± 0.3%), whereas F3 displayed a high opacity value (1.16 ± 0.3%) (*P* < 0.05), as presented in Table 1. This increase in opacity helps protect light‐sensitive foods during storage and packaging.^50^ Previous reports on polylactide and poly[(butylene adipate)‐*co*‐terephthalate)] films containing FA have shown that transparency decreases with the addition of FA.^66^ In another report, films developed using SA with FA incorporation demonstrated a higher opacity (11.28% for FA270) compared to the control without FA (3.63%).^51^

#### Mechanical properties

**Figure 2:**
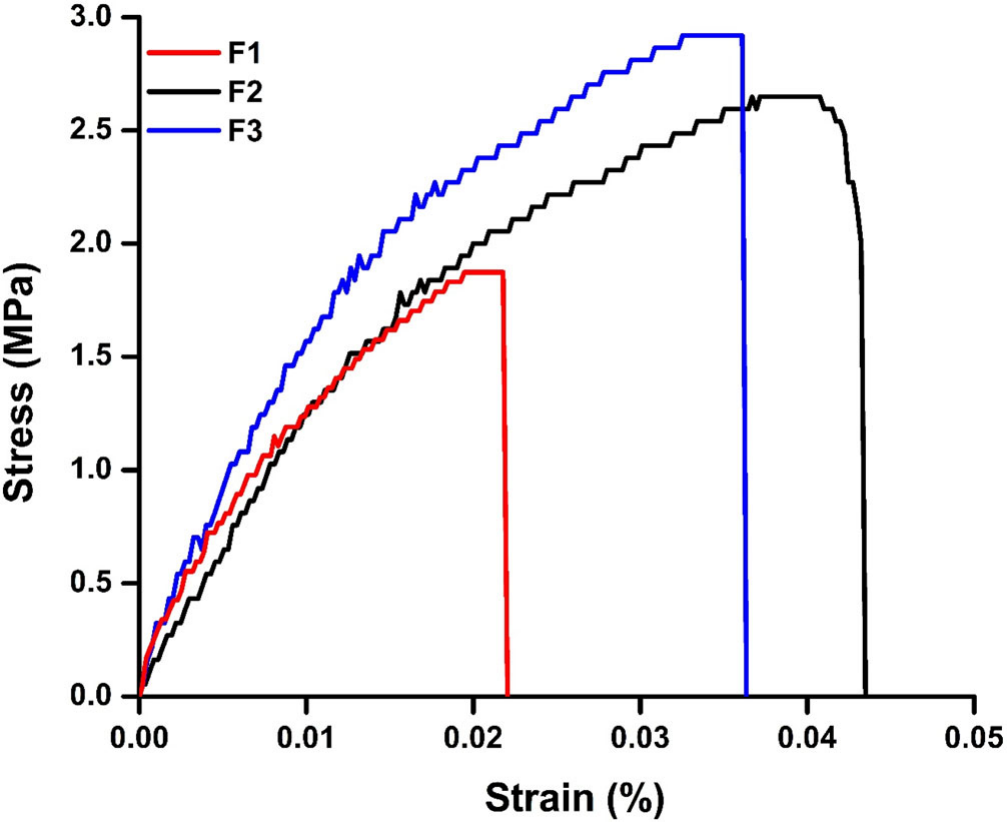
Stress–strain curves of active films, where strain (%) is represented on the *X*‐axis and stress (MPa) on the *Y*‐axis. F1, SA + P; F2, SA + P + 2.5 g L^−1^ FA; F3, SA + P + 5 g L^−1^ FA.

**Figure 3:**
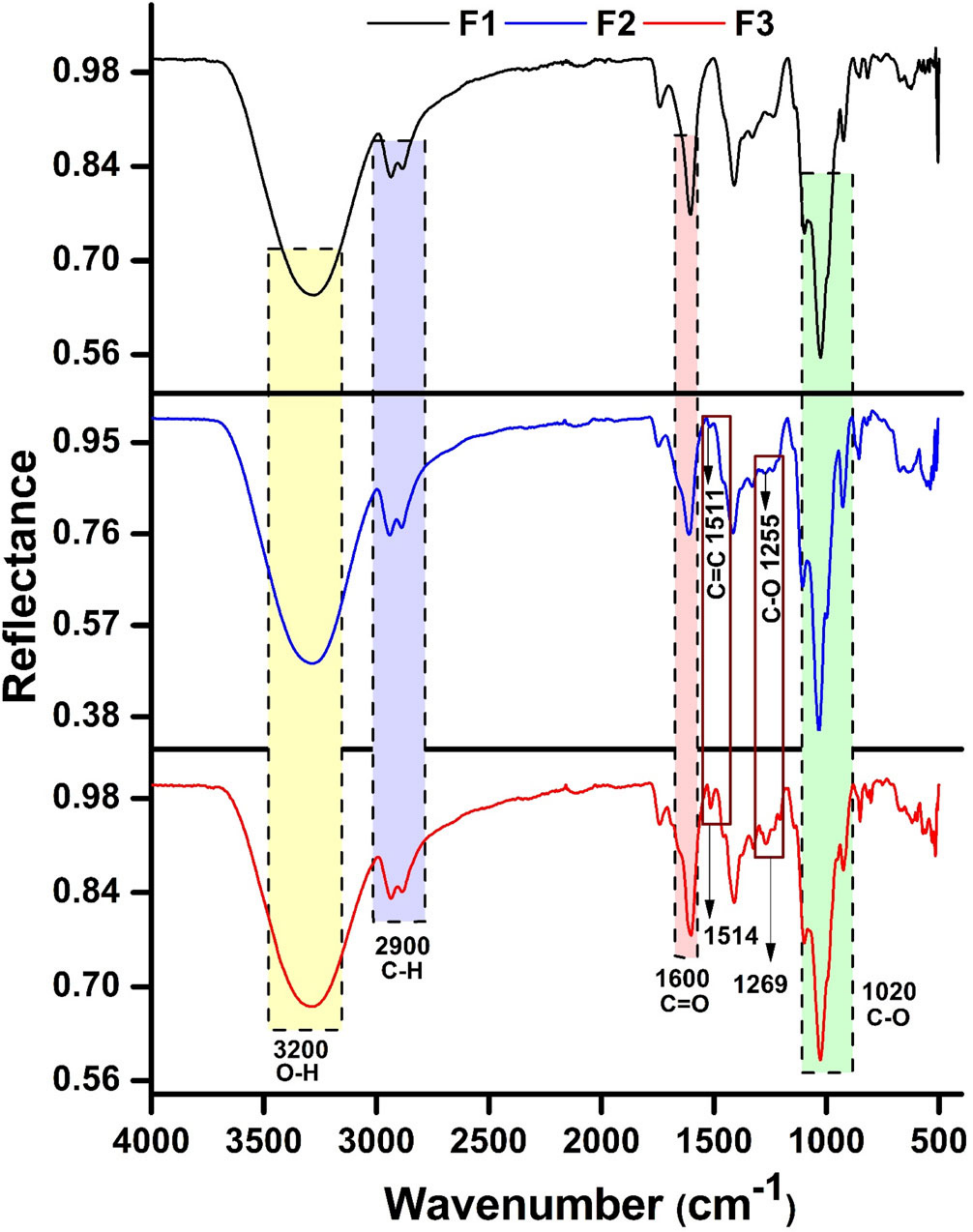
FTIR spectra of active films, where wavenumber (cm^−1^) is represented on the *X*‐axis and reflectance on the *Y*‐axis. F1, SA + P; F2, SA + P + 2.5 g L^−1^ FA; F3, SA + P + 5 g L^−1^ FA.

The mechanical properties of F1, F2, and F3 films, such as TS, EAB, and YM, are presented in Table 2, and the stress and strain curves are shown in Fig. 2. The addition of FA improved the film TS to 2.9 ± 0.8 MPa in F3 and 2.6 ± 0.5 MPa in F2, whereas the film with no FA (F1) exhibited a lower TS of 1.8 ± 0.5 MPa (*P* < 0.05). The increased TS (2.9 MPa in F3 *versus* 1.8 MPa in F1) and decreased SI (167 *versus* 214%) provide direct evidence of FA‐induced crosslinking and matrix densification (Table 1).^22^ The EAB of the F2 and F3 films was also high (0.043 ± 1.2% and 0.036 ± 1.2%, respectively) when compared to the F1 film (0.022 ± 0.9%) (*P* < 0.05), confirming the improved flexibility of FA‐incorporated films. These improved elastic properties were attributed to the interaction of FA with the film matrix, which enhances the polymer's structural mobility. In contrast, YM of the F2 film was less (60.5 ± 1.2 MPa) when compared to F1 (81.8 ± 1.4 MPa) and F3 (80.5 ± 1.4 MPa) (*P* < 0.05). The F3 film, with a high FA concentration, can form stronger, denser interactions with the film matrix, leading to a stiffer, more compact film. However, the moderate concentration of FA in the F2 film results in less efficient crosslinking with the film matrix, as the plasticizing effect increases with greater polymer chain spacing. This leads to reduced intermolecular interactions, which further decreases film stiffness, thereby improving flexibility, which was further supported by the increase in intensity of functional groups in FTIR spectra (F2 and F3) (Fig. 3).^67^ A similar trend in mechanical properties has been reported in a previous study, where FA of different concentrations (1%, 2%, and 10%) was incorporated into a collagen matrix.^67^

**Table 2:** Mechanical properties of active films

Film	TS (MPa)	EAB (%)	YM (MPa)
F1	1.8 ± 0.5^a^	0.022 ± 0.9^a^	81.8 ± 1.4^a^
F2	2.6 ± 0.5^b^	0.043 ± 1.2^b^	60.5 ± 1.2^b^
F3	2.9 ± 0.8^b^	0.036 ± 1.2^c^	80.5 ± 1.4^c^

F1, SA + P; F2, SA + P + 2.5 g L^−1^ FA; F3, SA + P + 5 g L^−1^ FA. Data are presented as mean ± SD, and all measurements were made in triplicate. Significant differences (*P* < 0.05) are represented by different letters according to Tukey's HSD test.

EAB, elongation at break; FA, ferulic acid; P, pectin; SA, sodium alginate; TS, tensile strength; YM, Young's modulus.

#### Antioxidant analysis

The study aims to integrate an antioxidant compound to enhance the AA of the SA–P film, based on the physicochemical interactions among food material, packaging system, and environmental conditions.^68^ Adding FA into the SA–P film matrix enhances its antioxidant properties, rendering the film both stable and active (Table 1). As the concentration of FA increases from 25 to 5 g L^−1^, the AA of the films increases significantly (*P* < 0.05) from 85 ± 1.1% to 90 ± 0.95%, while the control (F1) film shows the lowest AA effect at 30 ± 2.5%. Ascorbic acid was used as a standard control with AA of 93 ± 1.5%. These results suggest that incorporating FA into the polymer matrix improves AA, indicating the presence of a phenolic ring and an unsaturated side chain, which allows the formation of stable phenoxy radicals.^69^ A study on films based on ethylene vinyl alcohol containing FA showed a maximum scavenging activity of 70%, further supporting the above data.^68^

#### Release of FA from films

The release of phenolic compounds into the food simulant was determined from release studies conducted on films F1, F2, and F3. As the concentration of FA increases from 0 to 5 g L^−1^, the amount of phenolic compounds released significantly (*P* < 0.05) increases (0.42 ± 0.09 to 1.4 ± 0.05 mg g^−1^) (Table 1). This supports the enhanced antioxidant availability in F3 film (5 g L^−1^ FA), which delays the oxidation of high‐lipid‐containing foods.^24^ Furthermore, as FA is easily soluble in ethanol, it can release antioxidants more efficiently into ethanol‐rich simulants, which represent lipid‐rich foods.^70^ This leads to an increase in the shelf life of food products by reducing lipid oxidation. A similar trend for release studies has been reported in previous studies using various phenolic components to develop active packaging.^24^, ^70^, ^71^

#### Structural and morphological characteristics of films

##### FTIR spectroscopy

The FTIR analysis of the films (F1, F2, and F3) revealed differences in chemical composition and functional groups in the active film. The absorption band at 3200 cm^−1^ was related to an O—H stretching vibration (hydroxyl group), while the 1600 cm^−1^ peak represents the C=O stretching vibration (carbonyl group) of P. A similar range of P peaks has been reported in a previous study.^72^ SA was evident from characteristic peaks at 2900 cm^−1^, attributed to the C—H stretching vibration, and at 1020 cm^−1^, attributed to the C—O stretching vibration, which aligns with earlier findings.^33^ As the base matrix of the film is composed of SA and P, these peaks were clearly visible in the spectra of F1, F2, and F3 films (Fig. 3). The incorporation of FA in F2 and F3 films led to new peaks at 1511 and 1514 cm^−1^, indicating aromatic bonds of C=C, and at 1255 and 1269 cm^−1^, indicating C—O stretching (carboxylic acid). As the FA concentration increases from F2 to F3, the peak intensity increases. The presence of these interactions will further reduce the availability of hydrophilic groups, thereby further limiting the number of water‐binding sites.^64^ This observation further supports the reduction in the values of MC, SI, WS, and *a*_w_. The above results were further supported by a previous study of chitosan and poly(vinyl alcohol) films incorporated with FA.^73^ Based on the above results (physical, barrier, optical, mechanical, and AA), F3 showed superior performance; hence, further analyses were conducted on F1 and F3.

##### XRD analysis

**Figure 4:**
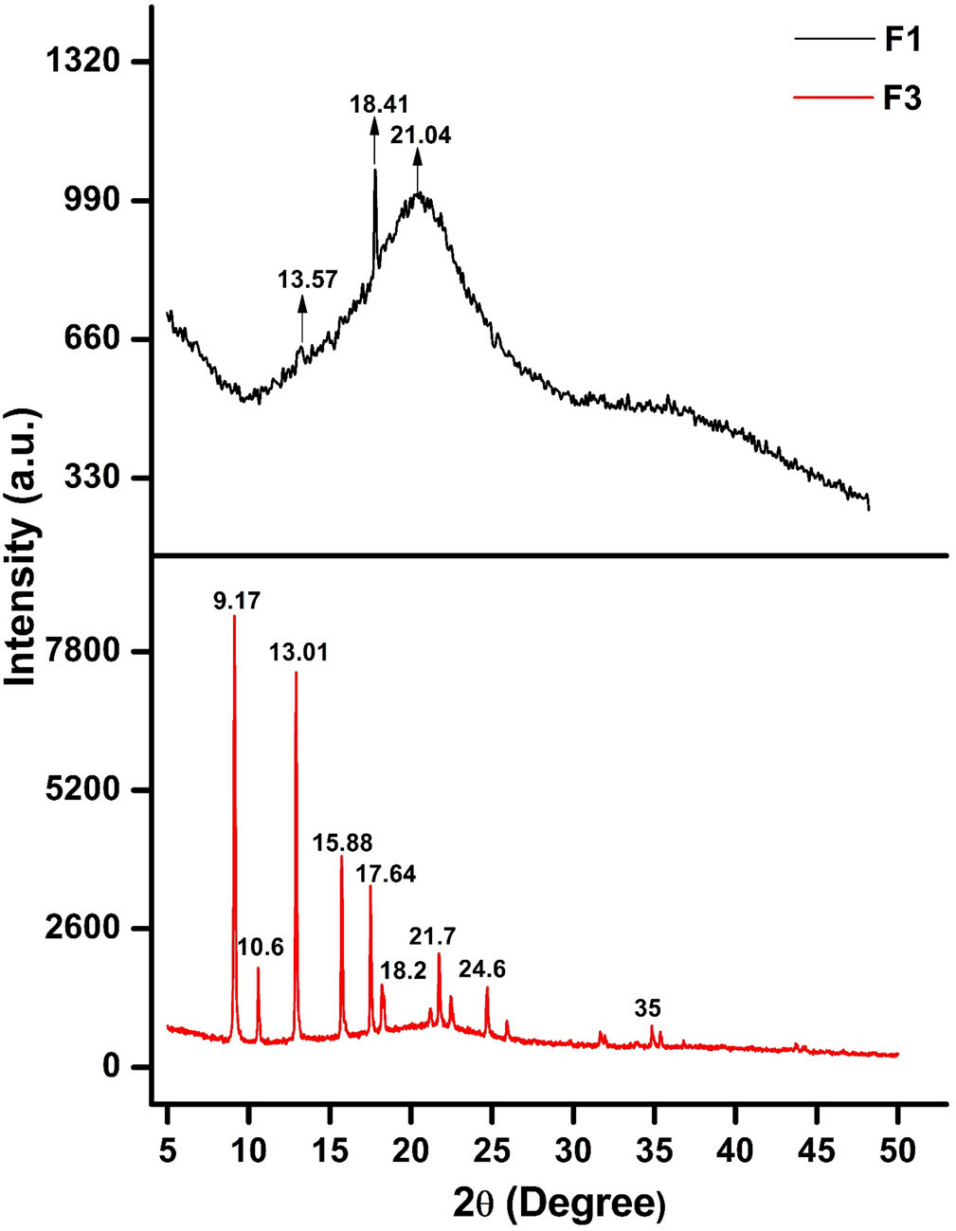
XRD patterns of active films, where the diffraction angle (2*θ*) is represented on the *X*‐axis and intensity (arbitrary units) on the *Y*‐axis. F1, SA + P; F3, SA + P + 5 g L^−1^ FA.

The crystallinity of the SA–P–FA matrix was studied using XRD, where the F1 film, without FA, and the F3 film, with FA, were compared (Fig. 4). The films F1 and F3 exhibited characteristic peaks of SA at 13.57°, 21.04° (F1), 13.01° and 21.7° (F3), indicating the semicrystalline nature. The characteristic peak of P was observed at 18.41° and 18.2° in F1 and F3 films, indicating its semicrystalline nature. These results were supported by previous studies on SA and P, which indicated peaks at a similar range.^33^, ^74^ The integration of FA in F3 film further enhances the film's characteristic diffraction pattern, which is attributed to crystalline compounds, exhibiting sharp peaks at 9.17°, 10.6°, 15.88°, 17.64°, 24.6°, and 35°. Similar diffraction enhancements have been reported in previous studies where FA was incorporated as an active component in films.^75^, ^76^ Furthermore, the degree of crystallinity of the F1 and F3 films was calculated, with crystallinity increasing from 47.8% to 73.8% for F1 and F3, respectively. This corresponds to the incorporation of FA into the F3 film, thereby increasing its crystallinity. These findings were consistent with a previous study that found that adding FA increased a film's crystallinity.^41^

##### FE‐SEM observations

**Figure 5:**
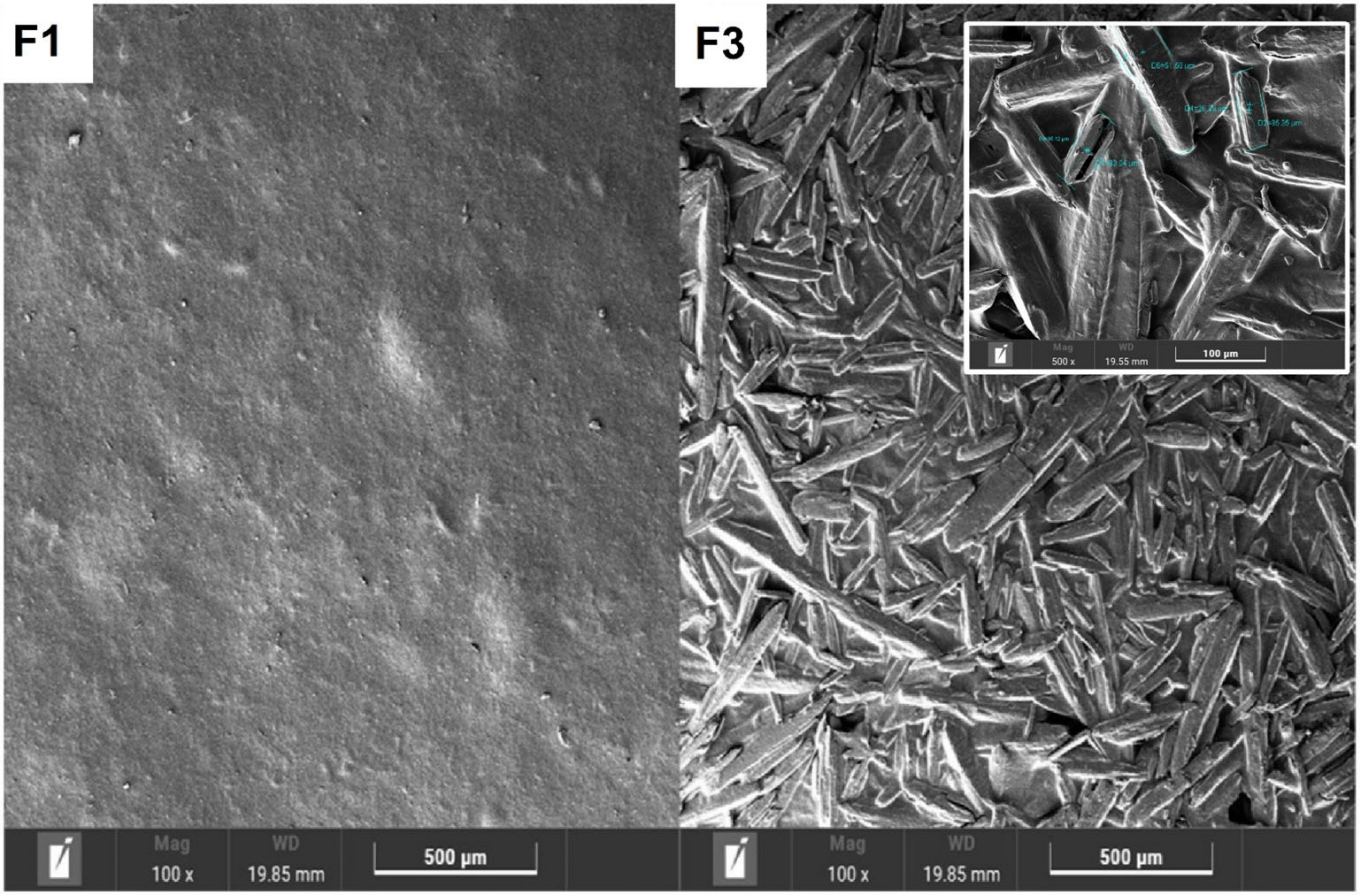
FE‐SEM images of active films and a magnified view of the F3 film, highlighting particle size. F1, SA + P; F3, SA + P + 5 g L^−1^ FA.

The F1 (SA + P) and F3 (SA + P + 5 g L^−1^ FA) films were examined using FE‐SEM analysis to investigate their surface morphology (Fig. 5). The F1 film exhibited a smooth and homogeneous surface with no discernible crystalline structures. In contrast, the F3 film containing FA displayed a longitudinal crystalline and heterogeneous structure, indicating the presence of FA embedded in the SA–P matrix. The particle size of FA is generally reported to be in the range of 10–190 μm.^77^ In the present study, the observed particle size distribution was found to be consistent with this reported range (Fig. 5). Similar observations were reported in a previous study, where the surface of the FA films exhibited visible particles of FA.^66^, ^67^

### Shelf‐life studies

**Figure 6:**
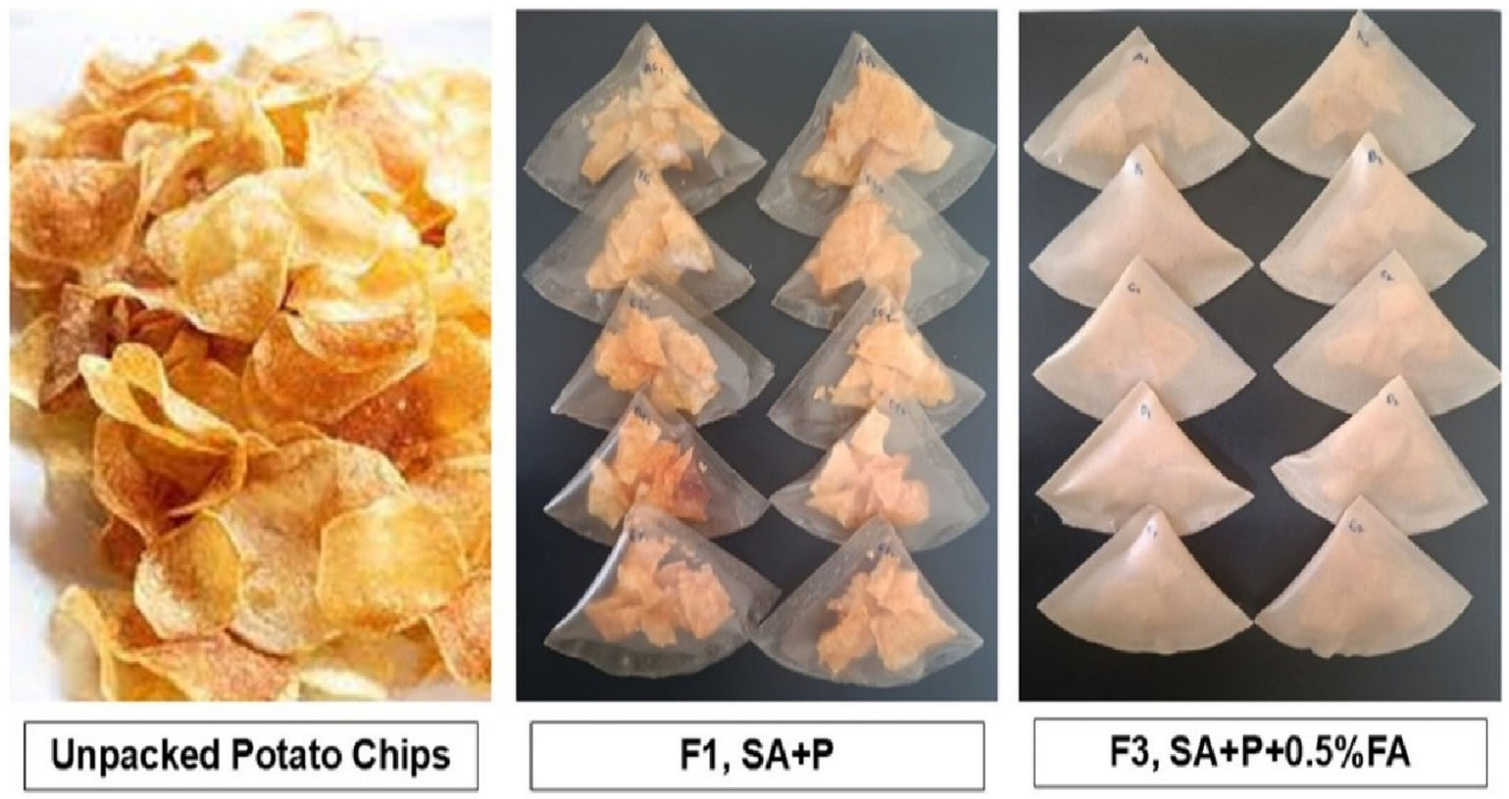
Packaging of potato chips. Unpacked chips without wrapping; F1, SA + P; F3, SA + P + 5 g L^−1^ FA.

Based on the above characterization, shelf‐life studies were conducted on potato chips wrapped with F3 film (SA + P + 5 g L^−1^ FA) and F1 film (SA + P) to assess the impact of FA, while control samples remained unpackaged (Fig. 6).

#### Moisture content

The increase in MC affects the texture and shelf life of the chips, making MC a crucial parameter for quality control. As potato chips are sensitive to moisture, it is essential to use a moisture‐resistant, highly selective packaging.^78^ In this study, unpacked chips exhibited high moisture levels when they reached day 10 (4.2 ± 0.4% to 16 ± 0.4%), which is considered very high. The F1 film with no FA showed comparatively less moisture (4 ± 0.5% to 11 ± 0.2%) in chips compared to unpacked chips, due to the presence of SA + P packaging. In contrast, the F3 film contains a high concentration of FA, i.e. 5 g L^−1^, which maintains the moisture of the chips better than the unpacked and F1 films until day 10 (4.2 ± 0.3% to 6.2 ± 0.4%) (*P* < 0.05) (Table 3). The reduced MC in chips packed in F3 was observed due to the higher concentration of FA, which contributes to a more compact film matrix with hydrophobic characteristics resulting from its nonpolar structural components.^79^, ^80^ A previous study also reported an increasing trend in MC, further supporting the results.^81^

**Table 3:** Moisture content, water activity, and peroxide value of potato chips

Films	Days	0	2	4	6	8	10
Unpacked	MC	4.2 ± 0.4^aA^	6 ± 0.3^aA^	7 ± 0.3^aA^	10 + 0.2^aA^	13 ± 0.3^abA^	16 ± 0.4^abA^
	WA	0.4 ± 0.07^aA^	0.5 ± 0.01^bA^	0.6 ± 0.04^bA^	0.67 ± 0.03^bA^	0.7 ± 0.04^bcA^	0.75 ± 0.02^bcA^
	Peroxide value	3.5 ± 0.7^aA^	8.5 ± 0.5^abA^	19.5 ± 0.8^bA^	30 ± 0.8^cA^	42 ± 0.5^cdA^	49 ± 0.7^cdA^
F1	MC	4 ± 0.5^aA^	4.5 ± 0.3^aB^	5 ± 0.2^aB^	6.4 ± 0.4^aB^	8.3 ± 0.5^abB^	11 ± 0.2^abB^
	WA	0.4 ± 0.04^aA^	0.49 ± 0.03^bA^	0.54 ± 0.04^bB^	0.58 ± 0.06^bB^	0.6 ± 0.05^bcB^	0.63 ± 0.02^bcB^
	Peroxide value	3.5 ± 0.7^aA^	5.5 ± 0.5^bB^	5.9 ± 0.5^bB^	6.5 ± 0.6^cB^	8.5 ± 0.7^cdB^	13 ± 0.8^cdB^
F3	MC	4.2 ± 0.3^aA^	4.4 ± 0.3^aB^	4.7 ± 0.2^aB^	5 ± 0.3^aC^	5.6 ± 0.6^abC^	6.2 ± 0.4^abC^
	WA	0.4 ± 0.03^aA^	0.44 ± 0.04^bB^	0.46 ± 0.01^bC^	0.5 ± 0.05^bC^	0.52 ± 0.06^bcC^	0.54 ± 0.04^bcC^
	Peroxide value	4 ± 0.6^aA^	4.5 ± 0.5^bC^	4.7 ± 0.7^bC^	5.2 ± 0.5^cC^	5.5 ± 0.8^cdC^	6.5 ± 0.6^cdC^

Unpacked, unpacked chips without wrapping; F1, SA + P; F3, SA + P + 5 g L^−1^ FA. Data are presented as mean ± SD, and all measurements were made in triplicate. Significant differences (*P* < 0.05) are represented by different lowercase letters (a, b, c, and d) within the same row (storage days) and different uppercase letters (A, B, and C) within the same column (between films on a given day) according to the Bonferroni test.

FA, ferulic acid; MC, moisture content; P, pectin; SA, sodium alginate; WA, water activity.

##### Water activity

The level of unbound water present in the sample triggers a chemical reaction, and microbial growth was monitored by measuring *a*_w_. On day 10, unpacked chips showed a high *a*_w_ value of 0.75 ± 0.02, while the chips packed in F1 film showed *a*_w_ of 0.63 ± 0.02, indicating high free water availability. In contrast, the chips packed in F3 films exhibited a low *a*_w_ of 0.54 ± 0.04 (*P* < 0.05) (Table 3), indicating less free water availability and a lower risk of spoilage. The chips packed in F3 film showed an *a*_w_ value below 0.6 threshold, which inhibits microbial growth.^58^ These results were further supported by a study in which apple discs were treated with SA and aloe vera gel containing FA, showing no significant difference in *a*_w_.^82^

##### Peroxide value

PV indicates the extent of oxidation in oils and fats; a high PV indicates increased rancidity, resulting in off‐odors and off‐flavors. Monitoring PV helps assess the freshness of potato chips, due to their high lipid content (*P* < 0.05) (Table 3).^83^ According to previous reports, oils with a PV below 10 mEq kg^−1^ are considered safe, whereas those with a value higher than 10 mEq kg^−1^are considered usable but rancid.^84^ In this study, the PV of unpacked chips reached 49 ± 0.7 mEq kg^−1^ at day 10, confirming that the unpacked potato chips exhibited rancidity. The chips packed in F1 film showed lower PV but still reached 13 ± 0.8 mEq kg^−1^ on day 10, which is undesirable for consumption. In contrast, the PV of chips packed in F3 film was very low (6.5 ± 0.6 mEq kg^−1^), indicating the absence of rancidity even after 10 days of storage. This enhanced stability of the films was observed due to the AA of FA, as shown in release studies, and to the film opacity resulting from FA incorporation, which acts as a light barrier.^45^ In a study where films were made using poly(lactic acid), thermoplastic starch and poly[(butylene adipate)‐*co*‐terephthalate)], along with the incorporation of chitosan and FA, when these films were applied to potato chips, they exhibited less PV compared to the control film without chitosan and FA.^42^

##### Antioxidant activity

**Figure 7:**
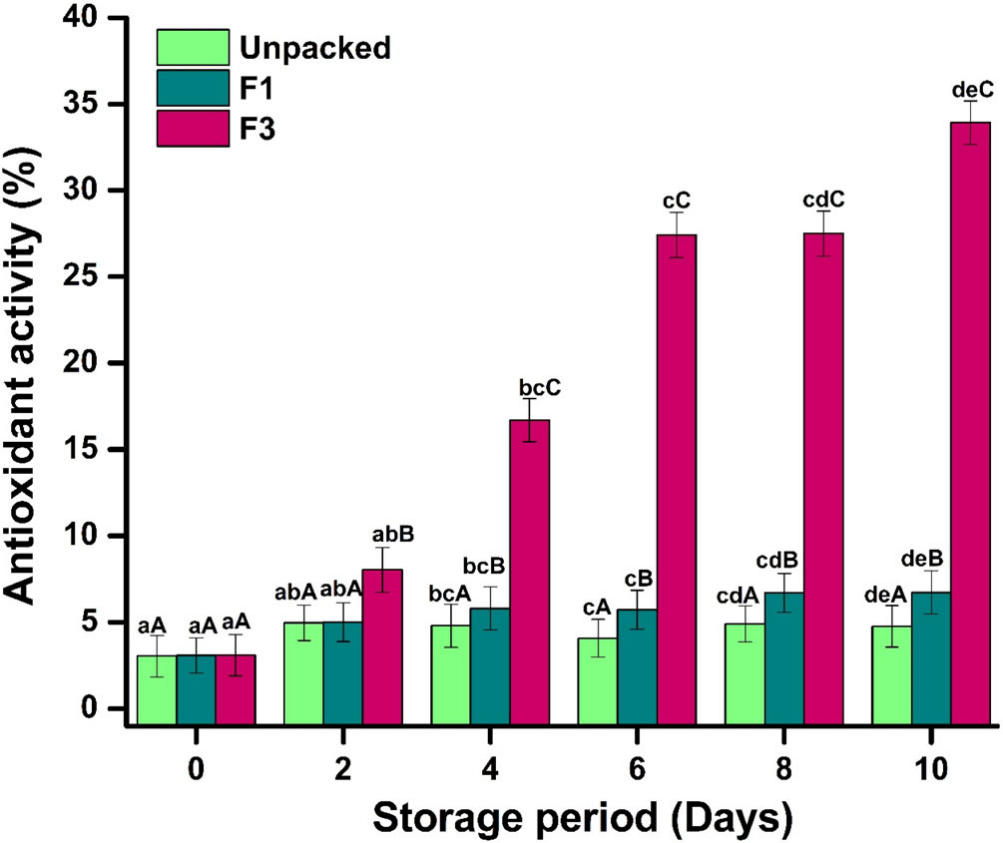
AA of potato chips packed in active films during a 10‐day storage period, where storage period (days) is represented on the *X*‐axis and AA (%) on the *Y*‐axis. Unpacked chips without wrapping; F1, SA + P; F3, SA + P + 5 g L^−1^ FA. The data are presented as mean ± standard deviation (SD), and all measurements were performed in triplicate. Significant differences (*P* < 0.05) are represented by different lowercase letters (a, b, c, d, and e) within the same row (storage days) and different uppercase letters (A, B, and C) within the same column (between films on a given day) according to the Bonferroni test.

Potato chips have a high lipid content and are highly susceptible to oxidation, which can be mitigated by incorporating natural antioxidants, such as FA, to reduce oxidation.^24^, ^85^ In this study, the chips packed in F3 film exhibited high AA (34 ± 1.25%) due to the presence of FA, which prevents oxidation of the chips. In contrast, the unpacked chips exhibited negligible AA (4.7 ± 1.2%), leading to oxidation of the chips due to their lack of external packaging (*P* < 0.05) (Fig. 7). These results were further supported by previous studies on apple slices and dried meat, which found that the incorporation of FA enhanced AA and minimized oxidative degradation.^24^, ^86^

##### Hardness/crispiness

**Figure 8:**
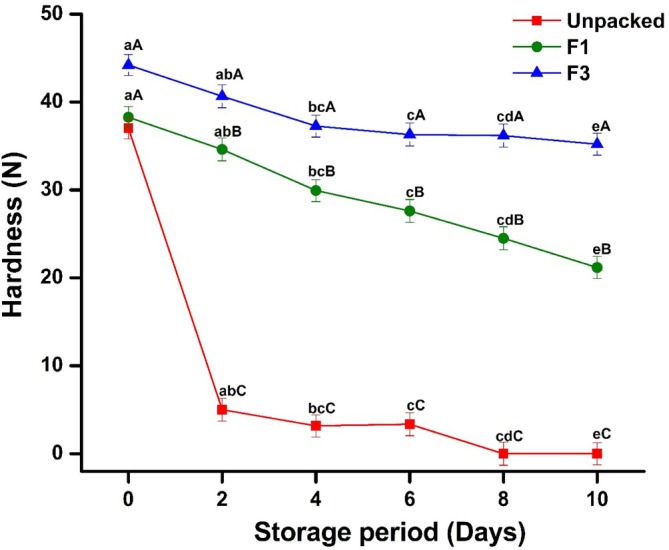
Hardness of potato chips packed in active films during a 10‐day storage period, where storage period (days) is represented on the *X*‐axis and hardness (N) on the *Y*‐axis. Unpacked chips without wrapping; F1, SA + P; F3, SA + P + 5 g L^−1^ FA. The data are presented as mean ± SD, and all measurements were made in triplicate. Significant differences (*P* < 0.05) are represented by different lowercase letters (a, b, c, d, and e) within the same row (storage days) and different uppercase letters (A, B, and C) within the same column (between films on a given day) according to the Bonferroni test.

The crispiness of potato chips is a key quality parameter, as it strongly affects their quality and consumer acceptability. The hardness/crispiness of unpacked potato chips decreased and was entirely lost by day 6 of the analysis (3.35 ± 1.3 N). In contrast, the hardness of chips packed in F3 film maintained their crispiness until day 10 (35.2 ± 1.2 N), compared to chips packed in F1 film (*P* < 0.05) (Fig. 8). The MC of potato chips can further support these results: as the MC increases, the crispiness decreases. The incorporation of FA into the SA–P matrix effectively reduces MC, which maintains the crispiness of potato chips for 10 days. This observation aligns with a previous report that found a reduction in chip hardness when packaging was damaged, underscoring the importance of an effective moisture barrier to preserve crispness.^87^ Since F3 film exhibited the most promising overall performance, its techno‐economic feasibility was analyzed and it was found to be cost‐effective (USD 0.129 per film) (Table S3).

##### Sensory evaluation

**Figure 9:**
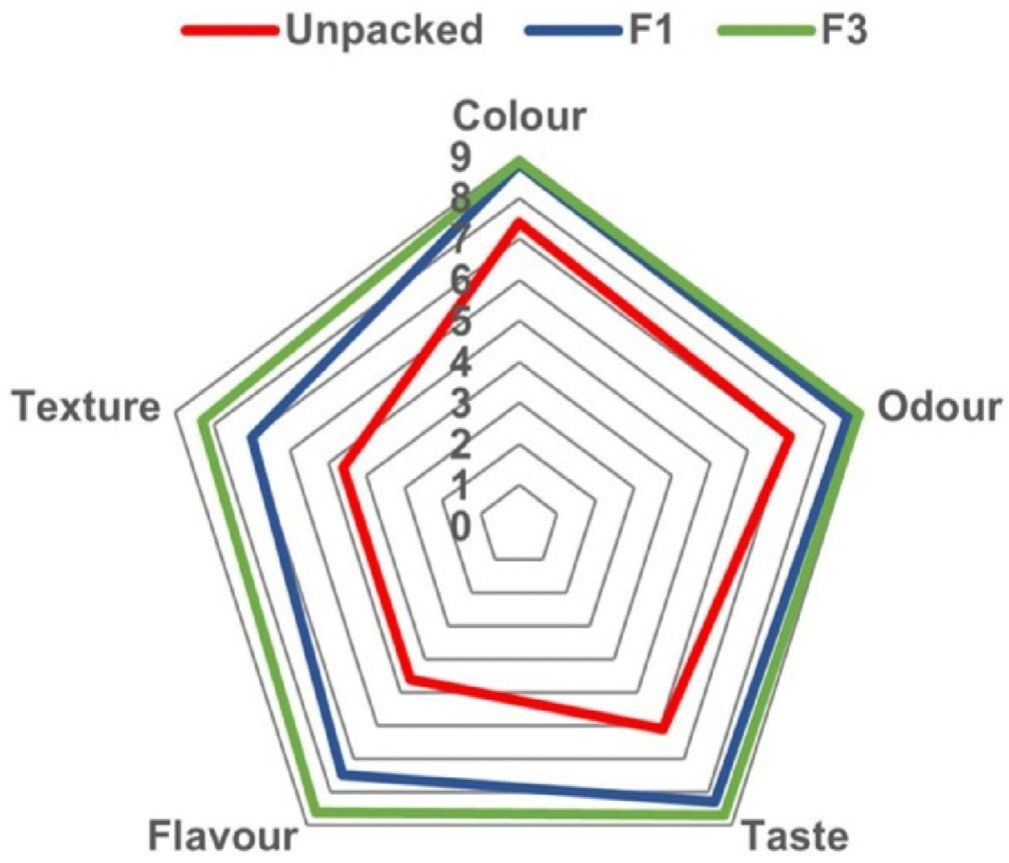
Sensory radar chart showing mean values of sensory attributes (color, odor, taste, flavor, and texture) of potato chips. Unpacked chips without wrapping; F1, SA + P; F3, SA + P + 5 g L^−1^ FA. The data are presented as mean ± SD, and all measurements were made in triplicate.

The sensory evaluation of potato chips was conducted on days 0, 2, 4, 6, 8, and 10 across various sensory attributes, including color, odor, taste, flavor, and texture. The overall mean values of all evaluated sensory attributes are presented in Fig. 9. According to the analysis, the chips packed in F3 are more desirable (*ca* 8.3 ‘like very much’) than those packed in F1 (*ca* 7 ‘like moderately’) or unpacked chips (≤5 ‘end of its acceptable sensory quality’). This was due to the presence of FA in the film, which prevents chips from absorbing moisture. The FA present acts as an excellent antioxidant, which prevents oxidation and quality deterioration in deep‐fried foods. As a result, incorporating FA into the SA–P matrix (F3) extends the shelf life of chips by maintaining their good color, odor, taste, flavor, and texture. Similar results were reported in a study in which FA was applied to dried meat, improving its shelf life while maintaining consumer acceptability.^86^

## CONCLUSION

FA was incorporated as a potential natural antioxidant to protect high‐lipid foods from oxidation‐induced deterioration. The incorporation of FA into the SA–P matrix not only improves the film's functional properties but also makes it an effective active packaging, representing a potential alternative to conventional materials. Among the developed formulations, the F3 film (SA + P + 5 g L^−1^ FA) has demonstrated enhanced hydrophobicity and antioxidant properties (90%), indicating its potential to increase the shelf life of potato chips. These enhanced properties of the F3 film are attributed to the incorporation of FA, which helps in intermolecular interactions within the SA–P matrix. This led to crosslinking, which simultaneously improved the film's mechanical, barrier, and antioxidant properties. In contrast, the F2 film was excluded due to its comparatively weak mechanical and functional properties. These films, when wrapped onto potato chips (unpacked, F1, and F3), showed that film F3 exhibited the highest inhibition towards oxidation up to 10 days of packaging. The chips packed in F3 film also maintained their MC, *a*_w_, PV, texture, and sensory properties, demonstrating consumer acceptability during storage. These findings demonstrate the potential of the packaging material by showing its applicability to other food products, particularly those rich in lipids. Further studies will focus on scaling up film production, evaluating antimicrobial activity during storage, and studying the applicability of other lipid‐rich foods. This study contributes towards reducing pollution and improving sustainability by incorporating this film, which helps mitigate the negative environmental impact typically associated with petroleum‐based food packaging.

## FUNDING INFORMATION

This work was supported by the University Grants Commission–Basic Scientific Research Start‐up Grant (UGC–BSR), Ref. No. F.30‐456/2018, and by Research Seed Grants from the Gandhi Institute of Technology and Management, Ref. No. F. No 2021/0087.

## CONFLICT OF INTEREST

The authors declare that there is no conflict of interest.

## AUTHOR CONTRIBUTIONS

SS: Data curation, analysis, writing, and editing of the manuscript. VGP: Funding, designing the experiments, data curation, analysis, overall supervision, writing, and editing of the manuscript. NN: Data curation, analysis, writing, and editing of the manuscript. AR: Data curation, analysis, and editing of the manuscript. NS: Data analysis and editing of the manuscript.

## Supporting information


**Table S1.** Comparison table summarizing previous FA‐based active films and their applications.
**Table S2.** Water vapor permeability of some common commercial packaging.
**Table S3.** Techno‐economic feasibility of F3 (SA, 13 g kg^−1^; P, 10 g kg^−1^; FA, 5 g L^−1^) film.

## Data Availability

The data that support the findings of this study are available from the corresponding author upon reasonable request.
