# Supplementary material for: Ferulic acid‐impregnated sodium alginate–pectin biopolymer film for active packaging and shelf‐life extension of potato chips
Source: J Sci Food Agric. 2026 Apr 21;106(11):6428–41. doi: 10.1002/jsfa.70672 (PMC13341072; doi:10.1002/jsfa.70672)
Supplement: Supplementary file 1 — Table S1. Comparison table summarizing previous FA‐based active films and their applications. Table S2. Water vapor permeability of some common commercial packaging. Table S3. Techno‐economic feasibility of F3 (SA, 13 g kg−1; P, 10 g kg−1; FA, 5 g L−1) film. [file JSFA-106-6428-s001.docx]

**Supporting Information**

**Ferulic Acid-Impregnated Sodium Alginate-Pectin Biopolymer Film for Active Packaging and Shelf-Life Extension of Potato Chips**

Shaik Sadiya^1^, Venkata Giridhar Poosarla^1^*, Navyasri Nandavarapu^1^, Aparna Ramadoss^1^, Nagaveni Shivshetty^1^

^1^Department of Life Sciences, GITAM School of Science, GITAM (Deemed to be University), Visakhapatnam, Andhra Pradesh, India, 530045.

*To whom correspondence should be addressed: [gpoosarl@gitam.edu](mailto:gpoosarl@gitam.edu)

**Table S1.** Comparison table summarizing previous FA-based active films and their applications.

| **Sl. no.** | **Biopolymer composition** | **Film preparation method** | **Application** | **Storage period** | **Observation** | **Reference** |
| --- | --- | --- | --- | --- | --- | --- |
| 1 | Polyethylene oxide, zein, FA | Electrospinning | Apple | 8 days at 4 ℃ | Packed apple slices showed reduced browning rate and weight loss, and preserved antioxidant activity compared to the control packaging | ^1^ |
| 2 | Chitosan, FA | Grafting | Pork | 8 days at 4 ℃ | Pork coated with ferulic acid grafted chitosan showed total viable counts, total volatile basic nitrogen values, pH values, thiobarbituric acid reactive substances, drip losses and better sensory characteristics (better color, and odor) | ^2^ |
| 3 | Aloe vera gel, SA, FA | - | Apple | 7 days at 5 ℃ | The coated fresh-cut apple discs demonstrated lower weight loss, browning, antioxidant activity, total phenolic content and FA and alginate-coated apples were effective in reducing *Listeria monocytogenes* | ^3^ |
| 4 | Carboxymethyl cellulose, polyethylene glycol, natamycin, FA | Solvent casting | Ras cheese | 11 days at 28 ℃ | Packed cheese had delayed the decline of sensory by maintaining the moisture, titratable acidity, hardness, and sensory characteristics | ^4^ |
| 5 | Poly(3-hydroxybutyrate-co-3-hydroxyvalerate), phenolic-rich extracts from rice straw, FA | Compression moulding | Pork | 16 days at 5 °C | Packed meat showed low pH and oxidation level, exhibited higher UV light blocking effect, maintained the color, and decreased the bacterial growth | ^5^ |

**Abbreviations:** FA, ferulic acid; SA, sodium alginate

**Table S2.** Water vapor permeability of some common commercial packaging.

| **Plastic**^6^ | **Water vapor permeability**  **(**10^–14^ g m^-1^.s^-1^.Pa^-1^) |
| --- | --- |
| Low-density polyethylene | 6.673-8.704 |
| High-density polyethylene | 1.741-3.482 |
| Polypropylene | 2.321-4.642 |
| Polyethylene terephthalate | 5.803-22.921 |
| Polystyrene | 11.315-45.552 |
| Poly(vinyl chloride) | 18.279 |
| Poly(vinylidene chloride) | 1.161 |
| Polyamide | 5.803-114.314 |

**Table S3.** Techno-economic feasibility of F3 (SA, 13 g kg^-1^; P, 10 g kg^-1^; FA, 5 g L^-1^) film.

| **S. No.** | **Base components** | **Price**  **(USD kg^-1^)** | **Quantity required to make one film** | **Cost (USD per film)** |
| --- | --- | --- | --- | --- |
| **1.** | Pectin | 68.32 | 1 g | 0.068 |
| **2.** | Sodium alginate | 20.61 | 1.3 g | 0.027 |
| **3.** | Ferulic acid | 9.49 | 0.5 g | 0.005 |
| **4.** | Glycerol | 19.14 | 1.5 mL | 0.029 |
| **5.** | Distilled water | - | 100 mL | - |
| **Total operating cost** | |  |  | **0.129 USD** |

**References:**

1. Huang X, Jiang W, Zhou J, Yu D-G and Liu H, The applications of ferulic-acid-Loaded fibrous films for fruit preservation. Polymers 14: 4947 (2022).

2. Wang G, Liu Y, Yong H, Zong S, Jin C and Liu J, Effect of ferulic acid-grafted-chitosan coating on the quality of pork during refrigerated storage. Foods (Basel, Switzerland) 10:

1374 (2021).

3. Nicolau-Lapeña I, Aguiló-Aguayo I, Kramer B, Abadias M, Viñas I and Muranyi P, Combination of ferulic acid with Aloe vera gel or alginate coatings for shelf-life prolongation of fresh-cut apples. Food Packag Shelf Life 27: 100620 (2021).

4. Abdin M, Naeem MA and Aly-Aldin MM, Enhancing the bioavailability and antioxidant activity of natamycin E235–ferulic acid loaded polyethylene glycol/carboxy methyl cellulose films as anti-microbial packaging for food application. Int J Biol Macromol 266: 131249 (2024).

5. Moll E and Chiralt A, Active PHBV films with ferulic acid or rice straw extracts for food preservation. LWT 228: 118115 (2025).

6. Bastarrachea L, Dhawan S, Sablani S, Engineering properties of polymeric-based antimicrobial films for food packaging: a review. Food Eng Rev 3, 79-93 (2011).
